# Racial Disparities in Obesity Prevalence in Mississippi: Role of Socio-Demographic Characteristics and Physical Activity

**DOI:** 10.3390/ijerph14030258

**Published:** 2017-03-03

**Authors:** Mina Qobadi, Marinelle Payton

**Affiliations:** Center of Excellence in Minority Health and Health Disparities, School of Public Health, Jackson State University, Jackson, MS 39213, USA; marinelle.payton@jsums.edu

**Keywords:** obesity, social disparities, socio-demographic characteristics, racial discrimination

## Abstract

Although the etiology of obesity is complex, social disparities are gaining attention for their contribution to obesity. The aim of this study was to estimate prevalence of obesity and to explore the associations between socio-demographic characteristics and obesity by race in Mississippi. Data from the 2014 Mississippi Behavior Risk Factors Surveillance System (BRFSS) were used in this study (*n* = 3794). Descriptive statistics, Chi-square tests and logistic regressions were conducted using SAS Proc. Survey procedures to account for BRFSS’s multistage complex survey design and sample weights. The overall prevalence of self-reported obesity was 37%. Multiple logistic regression model showed gender was the only variable associated with increased risk of obesity among blacks. Black females were more likely to be obese (Adjusted OR [aOR] = 2.0, 95% CI: 1.4–2.7, ref = male) after controlling for confounders. Among white adults, obesity was significantly associated with physical activity, gender, age and education levels. Those aged 25–44 years (aOR = 1.7, 95% CI: 1.1–2.6, ref ≥ 64 years), those were physically inactivity (aOR = 1.8, 95% CI: 1.4–2.5, ref = physically active) or had high school education (OR = 1.6, 95% CI: 1.2–2.3, ref = college graduate) or some college (aOR = 1.5, 95% CI: 1.2–2.3, ref = college graduate) were more likely to be obese; females (aOR = 0.8; 95% CI: 0.6–0.9, ref = male) and those aged 18–24 years (aOR = 0.50, 95% CI: 0.21–0.9, ref ≥ 64 years) were less likely to be obese.

## 1. Introduction

Obesity is often considered to be result of energy imbalance, which is defined as the balance between energy intake, energy expended through metabolism and physical activity and energy storage. Therefore, changes in body weight are expected when energy intake is not equal to energy expenditure over a certain period of time [1]. In the United States, the prevalence of obesity has increased rapidly during the past years. In 1990, no state had a prevalence of obesity greater than 15%, whereas in 2006, only four states had rates of less than 40% [2]. By 2010, 36 states had obesity rates of 25% or higher, and 12 of those had a prevalence rate of obesity equal to or greater than 30%. Nationwide today, more than one-third of adults are obese [3]. Mississippi currently has the second highest adult obesity rate in the nation (35.6%). According to the State of Obesity, the adult obesity rate in Mississippi has increased dramatically over the past years, from 15% in 1990 to 35.6% in 2015 and could reach 66.7% by 2030 [4,5].

It is well established that obesity has negative effects on our overall health, health care system and economy. Obesity is associated with a number of chronic diseases, including type II diabetes, hypertension, stroke, coronary heart disease, cancer and cancer-related mortality. Obesity-related medical treatment costs are estimated at 147 billion dollars, nearly 10% of all annual medical spending [6]. The annual nationwide productive costs of obesity (costs due to employees being absent from work and decreased productivity of employees while at work for obesity-related health reasons) range between $3.38 billion ($79 per obese individual) and $6.38 billion ($132 per obese individual) [7]. Overall, obese people spend 42% more on healthcare costs per year than normal weight people [6]. If obesity rates continue on their current trajectory, by 2030, obesity-related medical costs alone are estimated to rise by $48 to $66 billion a year in the U.S. [8]. In 2008, health-care costs directly related to obesity in Mississippi were $925 million ($441 per person) and if obesity levels hold at their 2008 rates, Mississippi could save an estimated $1017 per adult in healthcare costs by 2018 [9]. If the trend continues, health-care costs attributable to obesity will be $3.9 billion in 2018. If BMI is lowered by 5%, Mississippi could save 6.9% in health care costs, which provides a potential saving of $6.12 trillion by 2030 [10].

Eliminating health disparities, as a top public health priority in the United States, could be a solution for reducing obesity. One fundamental goal in the Healthy People 2020 is to achieve health equity and eliminate disparities. There is substantial research documenting the existence of sociodemographic and economic disparities in obesity; fewer studies have examined the disparities among different race groups. In addition, many studies have been done to identify socio-demographic overweight and obesity determinants at the national level, while little is known about the socio-demographic risk factors of overweight and obesity in Mississippi. Therefore, the aim of this study was to estimate the prevalence of obesity and to explore the associations between socio-demographic characteristics and obesity by race in Mississippi. Understanding the influence of these disparities will be critical to developing public policies and effective clinical interventions to prevent and treat obesity.

## 2. Methods

### 2.1. Sample and Survey Administration

The 2014 Mississippi Behavioral Risk Factor Surveillance System (BRFSS) data were used in this cross-sectional study. BRFSS is an ongoing, state-based survey conducted by state health departments in collaboration with the Center for Disease Control and Prevention (CDC). It uses a multistage cluster sampling design based on random-digit dialing (landline and cell phone) to select a representative sample from each state’s non-institutionalized civilian residents aged 18 years or older. Data from each state are weighted to compensate for unequal probabilities of selection, adjust for non-response and non-coverage to match the sample to the population and to make representative population**-**based estimates.

BRFSS questions are designed to gather information from adults on their health condition and health-related behaviors. The questionnaire has three parts: (1) the core component, (2) optional modules and (3) state-added questions. The CDC requires states to ask all questions on the core questionnaire of each respondent. Optional modules are sets of questions on specific topics that states may choose to use on their questionnaires. Individual states may develop their own questions and add these questions to their questionnaires. These state-added questions are not edited or evaluated by the CDC. Each year, the states and the CDC agree on the content of the core component and optional modules.

### 2.2. Outcome Variable

The outcome measure was obesity. We used BRFSS data on self-reported height and weight responses to calculate each respondent’s Body Mass Index (BMI), as a measure of obesity. Self-reported weight and height were assessed by asking respondents, “About how much do you weigh without shoes?” and “About how tall are you without shoes?” Obesity level was determined based on the following BMI criteria: underweight (BMI < 18.5), normal weight (18.5 ≤ BMI < 25), overweight (25 ≤ BMI < 30), obesity class I (≥30).

### 2.3. Explanatory Variables

Explanatory variables were socio-demographics such as gender, age, marital and employment status, race, income and education level. Healthy behavior in this study was leisure-time physical activity. We used two leisure-time physical activity categories (physically active or physically inactive). Adults who reported any physical activities or exercises, other than their regular job, such as running, calisthenics, golf, gardening, or walking for exercise during the past month was categorized as physically active. For socio-demographic characteristics, we used four age groups (18–24, 25–44, 45–64 and ≥65 years), four levels of education (less than high school, high school, some college and college graduate), three employment categories (employed, unemployed and retired), and two groups for marital status (married and unmarried). Taking into consideration that 98.5% of the population identified themselves as non-Hispanic black and non-Hispanic white, we used two groups for race (non-Hispanic white and non-Hispanic black) and excluded respondents of other races. Because a relatively large proportion (15.1%) of adults had “don’t know/refused/missing” responses for annual household income, we did not exclude these respondents from our analyses and we categorized annual household income as <$25,000, $25,000 to $49,999, ≥$50,000, or did not know/refused/missing. Participants were also excluded if they had unknown or missing data on obesity (*n* = 327), leaving a final analytic sample of 3794 adults. Missing data for other variables were verified and found to be less than 1%.

### 2.4. Statistical Analysis

We used χ^2^ tests to examine differences in obesity by socio-demographic characteristic and physical activity. Simple logistic regression was used to examine unadjusted bivariate association of obesity, sociodemographic characteristics and physical activity. Each predictor variable associated with obesity at *p* < 0.10 in the bivariate analyses was retained in the initial multiple logistic regression models [11]. Multiple logistic regression was conducted to estimate adjusted odds ratios for associations between the likelihood of obesity and socio-demographic and physical activity. Correlations between all independent variables were examined prior to inclusion in the models, in order to avoid problems associated with multi-collinearity. The sample weight variable was applied to all analyses to provide valid estimates for the civilian non-institutionalized adult population. All statistical analyses were performed using SAS 9.4 (SAS Institute, Cary, NC, USA) adjusting for the complex sample design of the BRFSS. Statistical tests were determined to be significant for *p* values < 0.05.

## 3. Results

The participants’ characteristics are presented in Table 1. The respondents were predominantly female, non-Hispanic white, unmarried, employed, aged 45–64 years old, and had some college education and less than $25,000 annual household income. Overall prevalence of self-reported obesity was 37% (95% Cl 34.8%–39.1%). Blacks, compared to white adults, were mostly females, unmarried, 25–44 years, obese and they had less education and income as well as higher unemployment rate and physical inactivity.

Our findings (Table 2) indicated there was a significant difference in rates of obesity by age, employment status, and physical activities in both races. The prevalence of obesity was significantly higher among adults aged 25–44 years, those who were unemployed and physically inactive. However, there were racial disparities in the prevalence of obesity by gender and education. Black females had higher rate of obesity compared to black males, while, there was no significant difference in rate of obesity by gender in whites. The prevalence of obesity was significantly lower in white adults with college degree while the prevalence of obesity was not significantly different by education levels in black adults.

Unadjusted and adjusted Odds Ratios (ORs) for the associations between obesity and social determinants are presented in Table 3. Model I (unadjusted model) showed that blacks had greater likelihood of obesity compared to whites (OR = 1.6; 95% CI: 1.3–1.9, ref = whites). In the unadjusted model, among black subgroup females (OR = 2.2; 95% CI: 1.6–3.0, ref = male), those who were unemployed (OR = 1.4; 95% CI: 1.1–2.0; ref = employed), and those who were physically inactive (OR = 1.4, 95% CI: 1.1–1.9, ref = physically active) were more likely to be obese, while among white subgroup adults aged 25–44 years (OR = 1.8, 95% CI: 1.3–2.5, ref ≥ 64 years), those with less than high school education (OR = 1.8, 95% CI: 1.1–2.6, ref = college graduate), and those who were physically inactive (OR = 1.8, 95% CI: 1.4–2.3, ref = physically active) were more likely to be obese.

Model II (adjusted model) showed that blacks were more likely to be obese compared to whites (Adjusted OR [aOR] = 1.6; 95% CI: 1.3–1.9, ref = whites), after controlling for the other variables. Among the black subgroup, gender was the only variable significantly associated with increased risk of obesity after controlling for the confounders. Our findings showed that black females were more likely to be obese (aOR = 2.0; 95% CI: 1.4–2.7, ref = male) after controlling for the confounders. Among the white subgroup, obesity was significantly associated with physical activity, gender, age and education levels after controlling for confounders. Among whites, those aged 25–44 years (aOR = 1.7, 95% CI: 1.1–2.6, ref = ≥64 years), those who were physically inactive (aOR = 1.8, 95% CI: 1.4–2.5, ref = physically active) or had high school education (aOR = 1.6, 95% CI: 1.1–2.3, ref = college graduate) or some college education (aOR = 1.5, 95% CI: 1.2–2.3, ref = college graduate) were more likely to be obese. Unlike black females, white females (aOR = 0.8; 95% CI: 0.6–0.9, ref = male) were less likely to be obese. Income and employment status had no association with obesity in both subgroups in the adjusted model.

## 4. Discussion

In 2014, more than one third of adults in Mississippi were obese. Similar to other studies, the prevalence of obesity was higher among females, blacks, adults aged 25–44 years, those with less than high school education, less than $25,000 annual income, unemployed adults, and those with no physical activity [3,12].

The reasons for the high rates of obesity in Mississippi could be due to differences in the food environment and marketing factors. Research showed that there is a strong association between advertisement exposure and poor diet. Exposure to marketing of calorie-dense foods can be a serious risk factor for obesity because it has been shown that ethnic minorities are highly responsive to this targeted marketing [13,14,15]. In addition to advertisement exposure, availability and affordability have been shown to be a key determinant of food choices and consumption [16]. Healthy foods increasingly cost more and fast food restaurants have become increasingly available and cheaper, possibly increasing the likelihood of obesity in Mississippi. Mississippi has the lowest annual household income in the nation, and low-income individuals may access junk food more easily than other nutrients, beverages and food because of differences in availability or prices. Furthermore, increased portion sizes and fast food consumption are typical dietary behaviors of people in Mississippi, which may increase the likelihood of obesity.

An additional key finding of this study is a considerable racial disparity in rates of obesity in Mississippi. Obesity continues to be higher among blacks, especially among black women [17,18]. Black adults reported higher rates of obesity than white adults at all levels of explanatory variables. Food and beverage marketing targets black Americans more frequently relative to whites, which may result in their higher levels of consumption of high-calorie foods and beverages and consequently, obesity. These industries have been using a variety of marketing tools to target black consumers through advertising, sales promotions, scholarship programs, and sponsorship of events within black communities, and the provision of employment opportunities. This heavy marketing influences individuals’ preferences, purchase requests, and consumption [19,20,21,22,23]. In addition, food consumption and dietary habits are deeply rooted in African American culture and history. Black families especially in southern states have their own traditional foods, often called soul foods, which are prepared with certain types of seasonings and ingredients. Despite containing healthy nutrients such as vegetables and grains, soul foods include high contents of fat, sugar and unhealthy amounts of salt that contribute to weight gain and obesity. Soul foods have a strong social component and are mainly served at family gatherings and holidays. Soul foods are often considered “good food”, compared with fast food by black families, indicating their skewed perception of healthy food choices. Additionally, lack of social support, the poor perceived taste of healthy foods, and the perception that eating healthy foods means giving up part of one’s culture and trying to comfort to dominant culture can justify the essential role of soul foods in food selection and eating behaviors among African Americans [24,25,26].

Our findings also showed that after controlling for confounders, gender, age, education, and physical activity were associated with increased risk of obesity among whites, while gender was the only variable significantly associated with increased risk of obesity among the black subgroup. One explanation could be that higher levels of social economic status do not translate into the same level of social opportunities and resources for blacks as for whites; as a result, middle-class blacks are more likely to live in disadvantaged neighborhoods than whites with similar socioeconomic status. Prior research indicated that black neighborhoods are more likely to have fast food restaurants and less likely to have fresh markets, parks, sidewalks and recreational resources [27]. Recent research suggests these community-level characteristics are associated with obesity independent of individual characteristics [28].

Our findings indicated that black women were twice as likely to be obese compared to black males, while white females were less likely to be obese compared to white males. Evidence has shown that obesity in women, especially those who are underrepresented such as black women, is associated with body image and weight perception. Prior research showed that black women are more prone to underestimate their weight due to different norms about body size and shape in their community compared to white women. Near half of overweight and obese black women do not classify their body size as overweight or obese. Black women are also more satisfied with their body size than whites, and see themselves more attractive than white women despite higher body mass. They also have fewer negative attitudes about qualities of overweight people than white women. In addition, many black men tend to prefer women with a heavier and curvaceous body type than white men, resulting in women’s lower engagement in weight loss activities [29,30]. Moreover, higher prevalence of obesity in black women might be associated with certain social factors including racial and residential discrimination. There is strong evidence that women who report more experiences of racial discrimination were more likely to be overweight or obese black women may choose food to cope with the disadvantage or negative feelings associated with residential discrimination and segregation [31]. There is also evidence that eating may reduce feelings of anxiety or stress [32,33]. Efforts are needed to identify and maximize resources that may reduce the negative effects of psychosocial factors on overall health and prevent obesity.

The strength of this study is that the analyses were based on a large, nationally-representative sample of United States adults. Results are thus generalizable on a population level and can be compared to other recent studies [34]. However, the findings in this report are subject to at least two limitations. First, estimates of obesity and explanatory variables were based on self-reporting, and respondents might not have accurately reported their height and weight; therefore, estimates might be either underestimated or overestimated. Second, these analyses were limited to adults in Mississippi, which limits the generalizability of the findings to the entire U.S. adult population. This study improves our understanding of adult obesity by focusing on variations by sociodemographic characteristics. Additional research is needed to understand the underlying risk factors of obesity among subgroups with higher prevalence.

## 5. Conclusions

Our findings indicated a high prevalence of obesity in Mississippi, which is consistent with national findings. The likelihood of obesity was greater among blacks, adults aged 25–44, and those with no physical activities, regardless of race. Accounting for race, gender was the only variable associated with increased risk of obesity among blacks. Our findings showed that black females are more likely to be obese after controlling for the confounders. Among white adults, obesity was significantly associated with physical activity, gender, age and education levels after controlling for confounders. Those aged 25–44 years, those with less physical activity or less education were more likely to be obese, and females and those aged 18–24 years were less likely to be obese. Eliminating disparities may reduce overweight and obesity and consequently, the risk of chronic diseases among U.S. adults. To reduce disparities in obesity, identifying variations in obesity by subpopulation groups and the development of targeted interventions such as providing access to more healthful alternatives to junk foods is needed. Additionally, effective community-based and faith-based programs must be developed, and those already found to be successful should inform legislation or policies regarding behavioral changes to reduce obesity and thus subsequent chronic health diseases by focusing on appropriate marketing, eating healthy food, being physically active, and stress management. Collaborative partnerships between health professionals and the community will also help identify needs and implement strategies rooted in the cultural context of the community. Further research should be done on the role of culture on dietary behaviors in this population.

## Figures and Tables

**Table 1:** Characteristics of total population, black and white adults, adjusted for sampling design: BRFSS, 2014.

	Total Percentage ^1^ (N ^2^)	Race		
		White	Black	
		Percentage ^1^ (N ^2^)	Percentage ^1^ (N ^2^)	*p*-Value ^3^
**Total**	3794	64.1 (2411)	35.9 (1383)	
**Gender**				0.03
Male	48.7 (1404)	50.5 (917)	45.6 (487)	
Female	51.3 (2390)	49.5 (1494)	54.4 (896)	
**Marital**				<0.0001
Married	49.2 (1812)	59.5 (1375)	30.7 (437)	
Unmarried	50.8 (1982)	40.5 (1036)	69.3 (946)	
**Age (years)**				<0.0001
18–24	12.2 (158)	11.9 (89)	12.8 (69)	
25–44	33.1 (720)	29.1 (369)	40.3 (351)	
45–64	34.3 (1462)	34.3 (857)	34.6 (605)	
≥65	20.3 (1454)	24.8 (1096)	12.3 (358)	
**Education**				<0.0001
<High School	18.4 (516)	15.1 (243)	24.4 (273)	
High School	29.9 (1208)	27.5 (719)	34.0 (489)	
Some College	33.3 (1005)	35.5 (680)	29.3 (325)	
College	18.4 (1065)	21.8 (769)	12.3 (296)	
**Income**				<0.0001
<$25,000	41.3 (1374)	30.1 (633)	61.2 (741)	
$25,000–$49,999	27.6 (883)	29.8 (603)	23.7 (280)	
≥$50,000	31.1 (1031)	40.1 (834)	15.0 (197)	
**Employment**				<0.0001
Employed	60.7 (1800)	61.9 (1157)	58.5 (643)	
Retired	18.2 (1218)	22.1 (910)	11.3 (308)	
Unemployed	21.1 (776)	16.0 (344)	30.2 (432)	
**Physical Activity**				<0.0001
Active	68.9 (2552)	72.8 (1669)	61.9 (883)	
Inactive	31.1 (1240)	27.2 (742)	38.1 (498)	
**BMI**				<0.0001
Normal	27.4 (1032)	30.0 (756)	22.9 (276)	
Overweight	35.6 (1394)	37.0 (930)	33.0 (464)	
Obese	37.0 (1368)	33.0 (725)	44.0 (643)	

^1^ Percentages are adjusted for the clustered sampling design of and unequal probability of selection into the data set. ^2^ Numbers are the absolute unadjusted numbers in each stratum. ^3^ Pearson chi-square test adjusted for the clustered sampling design of and unequal probability of selection into the data set.

**Table 2:** Prevalence of obesity by characteristics in the total population, blacks and white subgroups, BRFSS 2014.

	Obesity Prevalence					
	Total		White		Black	
	Percentage (N)	*p*-Value ^1^	Percentage (N)	*p*-Value ^1^	Percentage (N)	*p*-Value ^1^
**Total**	37.0 (1368)	-	-	-	-	-
**Gender**		0.02		0.21		<0.0001
Male	34.3 (453)		34.7 (286)		33.7 (167)	
Female	39.5 (915)		31.3 (439)		52.7 (476)	
**Marital**		0.4		0.06		0.3
Married	37.8 (693)		35.2 (434)		47.0 (205)	
Unmarried	36.1 (729)		29.8 (291)		42.7 (438)	
**Age (years)**		<0.0001		0.0004		0.0003
18–24	23.4 (36)		22.8 (19)		24.3 (17)	
25–44	43.6 (318)		39.2 (129)		49.2 (189)	
45–64	40 (589)		36.3 (299)		46.5 (290)	
≥65	29.2 (425)		26.0 (278)		40.6 (147)	
**Education**		0.01		0.01		0.9
<High School	40.2 (204)		35.8 (83)		45.1 (121)	
High School	38.4 (464)		34.6 (229)		43.9 (235)	
Some College	37.9 (369)		35.8 (218)		42.3 (151)	
College	29.7 (331)		24.6 (195)		46.1 (136)	
**Income**		0.003		0.3		0.3
<$25,000	41.8 (589)		35.2 (224)		47.7 (365)	
$25,000–$49,999	38.0 (330)		35.0 (204)		44.6 (126)	
≥$50,000	32.3 (309)		30.9 (221)		39.2 (88)	
**Employment**		<0.0001		0.0007		0.03
Employed	36.6 (649)		33.7 (351)		41.9 (298)	
Retired	28.2 (345)		25.3 (223)		38.0 (122)	
Unemployed	45.7 (374)		40.7 (151)		50.4 (223)	
**Physical Activity**		<0.0001		<0.0001		0.03
Active	33.1 (798)		29.4 (421)		40.8 (377)	
Inactive	45.6 (570)		42.7 (304)		49.4 (266)	
**Race**		<0.0001	-	-	-	-
White	33.0 (725)		-	-	-	-
Black	44.0 (643)		-	-	-	-

^1^ Pearson chi-square test adjusted for the clustered sampling design of and unequal probability of selection into the data set.

**Table 3:** Association between obesity and socio-demographic characteristics in the total population, black and white subgroups, BRFSS 2014.

	Unadjusted Model I ^a^			Adjusted Model II ^b^		
	Total	White	Black	Total	White	Black
**Gender**						
Female	1.2 (1.1–1.5)	0.9 (0.7–1.1)	2.2 (1.6–3.0)	1.1 (0.9–1.4)	0.8 (0.6–0.9)	2.0 (1.4–2.7)
Male	(ref)			(ref)	(ref)	(ref)
**Age (years)**						
18–24	0.7 (0.5–1.1)	0.8 (0.5–1.5)	0.5 (0.2–0.9)	0.4 (0.3–0.8)	0.5 (0.21–0.9)	0.4 (0.2–1.1)
25–44	1.9 (1.5–2.4)	1.8 (1.3–2.5)	1.4 (0.9–2.1)	1.6 (1.2–2.3)	1.7 (1.1–2.6)	1.6 (0.9–2.7)
45–64	1.6 (1.3–2.0)	1.6 (1.3–2.1)	1.3 (0.89–1.8)	1.3 (1.1–1.8)	1.3 (0.81–2.4)	1.3 (0.8–2.1)
≥64	(ref)	(ref)	(ref)	(ref)	(ref)	(ref)
**Education**						
<High school	1.6 (1.2–2.1)	1.8 (1.1–2.6)	0.96 (0.62–1.5)	1.1 (0.74–1.6)	1.4 (0.81–2.4)	0.78 (0.46–1.3)
High school	1.5 (1.2–1.9)	1.6 (1.2–2.2)	0.91 (0.62–1.3)	1.3 (0.96–1.7)	1.6 (1.1–2.3)	0.93 (0.59–1.5)
Some college	1.4 (1.1–1.8)	1.7 (1.3–2.3)	0.86 (0.56–1.3)	1.3 (1.0–1.7)	1.5 (1.2–2.3)	0.97 (0.62–1.5)
College graduate	(ref)	(ref)	(ref)	(ref)	(ref)	(ref)
**Income**				0.29	0.5	0.7
<$25,000	1.5 (1.2–1.9)	1.2 (0.88–1.7)	1.4 (0.90–2.3)	1.2 (0.88–1.6)	1.1 (0.0.76–1.7)	1.2 (0.73–2.1)
$25,000–49,999	1.3 (0.99–1.7)	1.2 (0.88–1.7)	1.3 (0.76–2.1)	1.2 (0.94–1.6)	1.2 (0.89–1.7)	1.2 (0.73–2.2)
≥$50,000	(ref)	(ref)	(ref)	(ref)	(ref)	(ref)
**Employment**				0.1	0.07	0.11
Retired	0.68 (0.55–0.8)	0.66 (0.51–0.86)	0.85 (0.58–1.2)	0.77 (0.57–1.1)	0.69 (0.48–1.00)	1.0 (0.60–1.7)
Unemployed	1.5 (1.2–1.8)	1.4 (0.96–1.9)	1.4 (1.0–2.0)	1.3 (0.95–1.7)	1.1 (0.75–1.7)	1.5 (0.99–2.3)
Employed	(ref)	(ref)	(ref)	(ref)	(ref)	(ref)
**Physical Activity**						
Inactive	1.7 (1.4–2.1)	1.8 (1.4–2.3)	1.4 (1.1–1.9)	1.5 (1.2–1.9)	1.8 (1.4–2.5)	1.3 (0.99–1.8)
Active	(ref)	(ref)	(ref)	(ref)	(ref)	(ref)
**Marital Status**						
Married	1.1 (0.89–1.3)	1.3 (0.98–1.7)	1.2 (0.88–1.6)	-	-	-
unmarried	(ref)	(ref)	(ref)	-	-	-
**Race**						
Black	1.6 (1.3–1.9)	-	-	1.4 (1.1–1.7)	-	-
White	(ref)	-	-	(ref)	-	-

^a^ Unadjusted Model; ^b^ Adjusted for cofounders excluding race and marital status.
